# Cardiac involvement in patients 1 year after recovery from moderate and severe COVID-19 infections

**DOI:** 10.3389/fcvm.2022.1009637

**Published:** 2022-10-26

**Authors:** Jinhan Qiao, Peijun Zhao, Jianyao Lu, Lu Huang, Xiaoling Ma, Xiaoyue Zhou, Liming Xia

**Affiliations:** ^1^Department of Radiology, Tongji Hospital, Tongji Medical College, Huazhong University of Science and Technology, Wuhan, China; ^2^MR Collaboration, Siemens Healthineers Ltd., Shanghai, China

**Keywords:** COVID-19, magnetic resonance imaging, cardiac involvement, late gadolinium enhancement, follow-up

## Abstract

**Background:**

Some patients suffered persistent cardiac symptoms after hospital discharge following COVID-19 infection, including chest tightness, chest pain, and palpitation. However, the cardiac involvement in these patients remains unknown. The purpose of this study was to investigate the effect of COVID-19 infection on the cardiovascular system after 1 year of recovery in patients hospitalized with persistent cardiac symptoms.

**Materials and methods:**

In this prospective observational study, a total of 32 patients who had COVID-19 (11 diagnosed as severe COVID-19 and 21 as moderate) with persistent cardiac symptoms after hospital discharge were enrolled. Contrast-enhanced cardiovascular magnetic resonance (CMR) imaging was performed on all patients. Comparisons were made with age- and sex-matched healthy controls (*n* = 13), and age-, sex- and risk factor-matched controls (*n* = 21). Further analysis was made between the severe and moderate COVID-19 cohorts.

**Results:**

The mean time interval between acute COVID-19 infection and CMR was 462 ± 18 days. Patients recovered from COVID-19 had reduced left ventricular ejection fraction (LVEF) (*p* = 0.003) and increased extracellular volumes (ECVs) (*p* = 0.023) compared with healthy controls. Focal late gadolinium enhancement (LGE) was found in 22 (68.8%) patients, mainly distributed linearly in the septal mid-wall or patchily in RV insertion point. The LGE extent in patients with severe COVID-19 was higher than that in patients with moderate COVID-19 (*p* = 0.009).

**Conclusion:**

This 1-year follow-up study revealed that patients with persistent cardiac symptoms, after recovering from COVID-19, had decreased cardiac function and increased ECV compared with healthy controls. Patients with COVID-19 predominately had a LGE pattern of septal mid-wall or RV insertion point. Patients with severe COVID-19 had greater LGE extent than patients with moderate COVID-19.

## Introduction

With the global pandemic caused by coronavirus disease 2019 (COVID-19), the World Health Organization (WHO) reported over 434 million cases and 5.9 million deaths as of February 2022 (1). In addition to respiratory system ailments, multiple extra-pulmonary injuries were identified in patients with COVID-19 (2). Acute cardiac injury was identified in histopathologic tissue sections in autopsy cases (3, 4), which included inflammatory infiltrates of interstitial mononuclear cells and small arterial obliteration due to vascular wall inflammation.

Given the potential risks of subendocardial myocardial biopsy, its application in COVID-19 survivors with suspected cardiac involvement is limited. Fortunately, cardiovascular magnetic resonance (CMR) imaging has allowed non-invasive investigation of myocardial injury by providing heart tissue characterization *in vivo*. Therefore, it is a valuable tool for detecting myocardial edema and myocarditis-like scars in patients with COVID-19 (5, 6). COVID-19-induced cardiac insults include cardiac edema in elite athletes 1-month post-diagnosis, myocardial edema, fibrosis, impaired right ventricular function in hospitalized patients 2 months following diagnosis, and fibrosis in patients with COVID-19 at the 3-month follow-up (7–9). Persistent cardiovascular abnormalities were not more common in asymptomatic patients 6 months after recovery (10). However, for patients with persistent chest pain or palpitations, the status of cardiac involvement after 1 year of recovery remains unknown (11, 12). Additionally, the correlation between cardiac involvement and severity of acute illness is controversial (7, 13).

The purpose of this study was to investigate the presence of cardiac injury by CMR in patients with persistent cardiac symptoms, namely chest tightness, chest pain, and palpitation, 1 year after recovery and to compare differences between subgroups of patients with moderate and severe COVID-19.

## Materials and methods

### Study design and participants

This prospective observational study was performed between April and June 2021. Inclusion criteria were: (1) patients with confirmed COVID-19 by RT-PCR on nasopharyngeal swab during hospitalization between January and March 2020; (2) patients volunteered for contrast-enhanced CMR examination; (3) patients suffered from persistent cardiac symptoms such as palpitation, chest pain or chest tightness after hospital discharge (8). Exclusion criteria included: (1) history of previous heart disease including myocarditis, cardiomyopathy/heart failure (dilated, hypertrophic, non-ischemic, ischemic), uncontrolled hypertension, coronary artery disease or prior myocardial infarction, moderate to severe valvular dysfunction, valve replacements, arrhythmias (such as atrial fibrillation, atrial flutter, or ventricular tachycardia), congenital heart disease; (2) cardiac symptoms present before COVID-19 infection or without persistent cardiac symptoms; (3) unwilling and/or unable to undergo contrast-enhanced magnetic resonance imaging; or (4) poor image quality due to severe arrhythmia (atrial fibrillation, premature ventricular beats, etc.) or inability to hold breath. Clinical, demographic characteristics, cardiac symptoms and blood test results were recorded on the day of CMR examination (12). Study patients were classified into moderate and severe COVID-19 subgroups according to the World Health Organization criteria (14, 15). The diagnostic criteria for patients with severe COVID-19 pneumonia were the presence of one of the following during hospitalization: respiratory rate > 30 breaths/min; severe respiratory distress; or SpO2 ≤ 93% on room air according to the WHOs interim guidance and the fifth edition of the “Pneumonia Diagnosis and Treatment Plan for New Coronavirus Infection” in China (15, 16). The remaining patients in our study who required hospitalization for observation and supportive care were defined as the moderate subgroup (14). This study was approved by the local ethics committee of our institution (2021-S114). All participants provided written informed consent.

Comparisons were performed with age- and sex-matched controls (healthy controls; *n* = 13), which were selected from a database in our institution of healthy subjects without systemic inflammation or cardiovascular disease. Besides, we included risk factor-matched controls to minimize the influence of cardiovascular risk factors on CMR characteristics, considering that some patients with COVID-19 had hypertension, diabetes, or hyperlipidemia. Age, sex, and cardiovascular risk factors (hypertension, diabetes, and hyperlipidemia) were matched between risk factor-matched controls and patients with COVID-19 as described in previous studies by Puntmann et al. (17) and Gao et al. (18). Comparisons were made with cardiovascular risk factor-matched patients (risk factor-matched controls; *n* = 21), who were examined in our institution before 2020.

The echocardiography and electrocardiogram (ECG) of the patients from different hospitals were assessed according to the reports. The severity of valvular regurgitation was assessed based on the echo gradient, color flow jet, continuous wave signal of jet, or effective regurgitant orifice area. Enlarged left atrium was assessed based on the maximum anteroposterior diameter of the left atrium (≥3.5 cm) and hypertrophic septum based on the maximum thickness of septum (≥1.0 cm). ST-segment elevation was defined as after the J point in 2 contiguous leads with the cutoff points of ≥0.2 mV in men and ≥0.15 mV in women in leads V2–V3, and/or ≥0.1 mV in other leads. ST-depression was defined as ≥0.1 mV at 80 ms from the J point, asymmetrical T-wave inversion ≥0.1 mV deep in 2 or more leads except aVR. QTc prolongation was defined as ≥450 ms for male and ≥470 ms for female (19).

### Cardiovascular magnetic resonance data acquisition

CMR was performed on a 3T MR scanner (MAGNETOM Skyra, Siemens Healthcare) using an 18-channel body coil combined with the spine coil. The exam protocols were (8): short-axis and long-axis cine, native T1/T2 mapping, late gadolinium enhancement (LGE), and post-contrast T1 mapping. Cine images were acquired by steady-state free precession (SSFP) with the following parameters: echo time (TE) = 1.4 ms; repetition time (TR) = 3.5 ms; flip angle (FA) = 55°; slice thickness = 8 mm; field of view (FOV) = 360 × 360 mm^2^, matrix = 256 × 256. Modified Look-Locker inversion recovery sequences with protocol 5b(3b)3b and 4b(1b)3b(1b)2b were used to obtain native and post-contrast T1 mapping, respectively. Parameters were as follows: TE = 1.2 ms; TR = 3.8 ms; FA = 35°; slice thickness = 8 mm; FOV = 340 × 340 mm^2^; matrix = 224 × 224. T2 mapping, a validated sequence for measurement of myocardial edema, was generated using a T2-prepared bSSFP sequence. Parameters were as follows: TE = 1.41 ms; TR = 3.3 ms; FA = 12°; slice thickness = 8 mm, matrix = 224 × 224, with three different T2 prepared time: 0, 24, 55 ms. LGE imaging was acquired 10–15 min after administration of gadobenate dimeglumine (0.2 ml/kg of Multihance, Bracco Diagnostics), using a phase-sensitive inversion recovery sequence with segmented FLASH readout and breath-holding. Imaging parameters were as follow: TE = 1.2 ms; TR = 5.2 ms; FA = 55°; slice thickness = 8.0 mm, matrix = 256 × 192. Post-contrast T1 mapping was performed 15–20 min after administering the contrast agent.

### Cardiovascular magnetic resonance images analysis

All CMR images were evaluated on CVI 42 (version 5.13, Circle Cardiovascular Imaging). The left ventricular (LV) myocardium was divided into 16 American Heart Association (AHA) segments. All LGE lesions were assessed by two radiologists independently (JQ and PZ with 3 and 5 years of CMR diagnosis experience, respectively), to obtain their localization (septal, lateral wall, and right ventricular (RV) insertion point, distribution (linear, patchy, and diffuse), and pattern (subendocardial, mid-wall, and subepicardial) (10, 20–22). Discrepancies between the two readers were adjudicated by a senior observer (LX with 20 years of CMR diagnosis experience). The endo- and epicardial contours on LGE images were manually delineated. The myocardial lesions in LGE images were defined as signal intensity > 3 standard deviations above the mean signal intensity of the remote reference myocardium (10). In LGE-positive patients, LGE ratios were calculated according to the LGE volume and total LV myocardium volume.

LV morphological and functional parameters, including cardiac volumes, mass, function, and strain, were obtained using the automated endocardial and epicardial contours detection and manually corrected if required. Global T1 and T2 values were measured by manually delineating the epicardium and endocardium of the whole LV myocardium region on T1 and T2 maps, which included LGE-positive regions. Extracellular volume (ECV) fraction was derived by native T1 and post-contrast T1 of the blood pool and myocardium (5). Hematocrit was measured within three days before CMR scanning.

### Statistical analysis

All statistical analysis was performed using SPSS Statistics 22 (IBM). Continuous data are presented as means (standard deviation) and categorical variables as counts (percentages). Normality distribution of continuous variables was tested using Shapiro–Wilk test. Comparisons were made between risk factor-matched controls and COVID-19 patients, healthy controls and COVID-19 patients, healthy controls and risk factor-matched controls. Student’s *t*-test was used for normally distributed data, and Mann–Whitney *U* test was used for non-normally distributed parameters. Proportions of categorical variables were conducted by χ^2^ and Fischer exact tests. Further comparisons were conducted between severe and moderate COVID-19. The agreement of LGE location, distribution, and pattern between the two readers was evaluated by kappa analysis (kappa > 0.80 was considered excellent agreement). All tests were 2-tailed, and *p* < 0.05 was considered statistically significant.

## Results

### Patient characteristics

**FIGURE 1:**
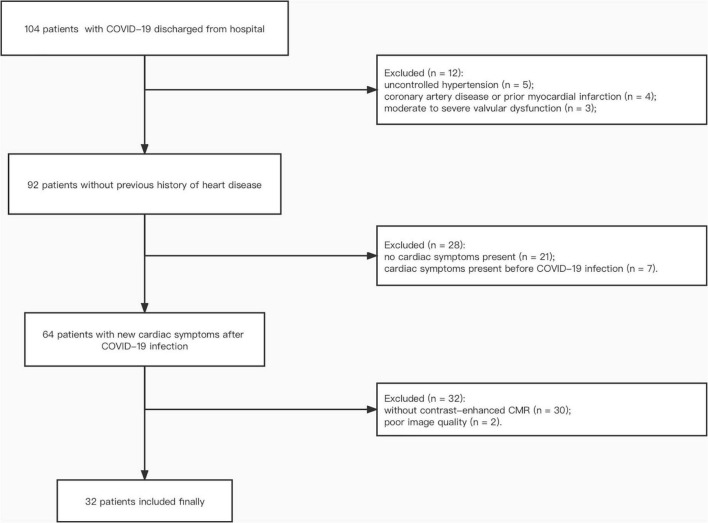
Participant flow diagram. COVID-19, coronavirus disease 2019; CMR, cardiac magnetic resonance.

A total of 32 patients who suffered from persistent cardiac symptoms after discharge from the hospital following COVID-19 infection were recruited and underwent enhanced CMR (Figure 1). These patients were treated at different hospitals in Hubei province, including 15 hospitals in Wuhan and 4 hospitals outside Wuhan city. The mean time interval from onset of acute infection to CMR examination was 462 ± 18 days. Baseline characteristics are shown in Table 1. Seven (21.9%) patients were male, and 11 (34.4%) were diagnosed with severe COVID-19 during hospitalization. There were no differences in the prevalence of hypertension, diabetes, and hypercholesterolemia between patients and risk factor-matched controls (*p* > 0.05).

**TABLE 1:** Demographic and clinical information of all participants.

	Healthy controls (*n* = 13)	Risk factor-matched controls (*n* = 21)	COVID-19 patients (*n* = 32)
Age (years)	45.6 ± 10.9	51.3 ± 8.0	48.7 ± 10.5
Male, *n* (*%*)	7 (53.8)	7 (33.3)	7 (21.9)
Time between acute COVID-19 infection and CMR (days)	–	–	461.9 ± 17.9
BMI (kg/m^2^)	22.8 ± 3.9	24.7 ± 4.3	24.0 ± 2.8
Systolic BP (mmHg)	116.7 ± 9.4	125.3 ± 15.3	118.7 ± 13.0
Diastolic BP (mmHg)	75.0 ± 9.6	78.5 ± 14.1	77.4 ± 11.7
Smoke, *n* (*%*)	1 (7.7)	4 (19.0)	5 (15.6)
Drink, *n* (*%*)	1 (7.7)	1 (4.8)	3 (9.4)
Hypertension, *n* (*%*)	0 (0)	6 (28.6)	7 (21.9)
Diabetes, *n* (*%*)	0 (0)	4 (19.0)	4 (12.5)
Hypercholesterolemia, *n* (*%*)	0 (0)	6 (28.6)	10 (31.3)^#^
Hct (%)	38.5 ± 3.0	39.2 ± 5.3	40.9 ± 3.5
Elevated troponin, *n* (*%*)^a^	0 (0)	1 (4.8)	11 (36.7)^#^^$^
Abnormal echocardiography, *n* (*%*)^b^	0 (0)	7 (33.3)*	11 (37.9)^#^
Abnormal electrocardiogram, *n* (*%*)^c^	0 (0)	7 (33.3)*	14 (50.0)^#^

Continuous data are presented as mean ± SD and categorical variables are summarized as percentage in parentheses.

COVID-19, coronavirus disease 2019; CMR, cardiovascular magnetic resonance; BMI, body mass index; BP, blood pressure; Hct, hematocrit.

^a^Troponin test was performed in 30 patients with COVID-19.

^b^Echocardiography was performed in 29 patients with COVID-19.

^c^ECG was performed in 28 patients with COVID-19.

*p < 0.05, healthy controls vs. risk factor-matched controls.

^#^*p* < 0.05, healthy controls vs. COVID-19 patients.

^$^*p* < 0.05, risk factor-matched controls vs. COVID-19 patients.

All patients reported one or more cardiac symptoms after discharge from the hospital, which included palpitation (*n* = 8, 25.0%), chest tightness (*n* = 21, 65.6%) and chest pain (*n* = 11, 34.4%). During hospitalization, 30 patients underwent troponin testing, of which 11 had elevated levels. Among the 29 COVID-19 patients with echocardiography, 7 (24.1%) showed mild mitral and/or tricuspid regurgitation, 3 (10.3%) had enlarged left atrium (3.7 ± 0.1 cm, anteroposterior diameter of the left atrium < 3.5 cm was deemed as normal), and 1 (3.4%) had a maximum thickness of septum of 1.2 cm (<1.0 cm was deemed as normal). ECG was performed on 28 patients, of which 14 were abnormal: sinus arrhythmia (*n* = 1, 3.6%), premature ventricular contractions (*n* = 1, 3.6%), T wave inversion (*n* = 4, 14.2%), ST-segment depression (*n* = 3, 10.7%), ST-segment elevation (*n* = 1, 3.6%), complete left bundle branch block (*n* = 1, 3.6%), and right bundle branch block (*n* = 3, 10.7%), QTc prolongation (*n* = 1, 3.6%). At follow-up, all troponins returned to normal levels. Six cases had persistent abnormal echocardiography.

The three tests (troponin test, echocardiography, and ECG) were performed on all controls. In the risk factor-matched control group, seven had abnormal echocardiography, including left ventricular wall thickening (*n* = 4, 19.0%), left atrium enlargement (*n* = 1, 4.8%), mild mitral and/or tricuspid regurgitation (*n* = 2, 9.5%). In addition, one subject had elevated troponin and seven had abnormal ECGs: ST-segment depression (*n* = 1, 4.8%), ST-segment elevation (*n* = 3, 14.3%), right bundle branch block (*n* = 2, 9.5%), and QTc prolongation (*n* = 1, 4.8%).

### Cardiovascular magnetic resonance analysis

**FIGURE 2:**
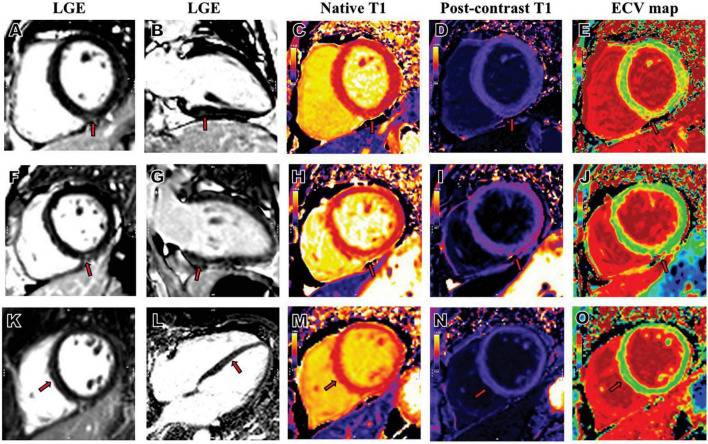
Representative LGE, native T1, post-contrast T1and ECV map images from patients who had COVID-19. A 34-year-old female patient with severe COVID-19, whose RV insertion point showed positive LGE in short axis **(A)**, 2-chamber long-axis **(B)**, increased native T1 **(C)**, decreased post-contrast T1 **(D)** and increased ECV **(E)**. A 50-year-old female patient with severe COVID-19, whose inferior mid-wall showed positive LGE in short axis **(F)**, 2-chamber long-axis **(G)**, increased native T1 **(H)**, decreased post-contrast T1 **(I)** and increased ECV **(J)**. A 46-year-old male patient with severe COVID-19, whose septal mid-wall showed positive LGE in short axis **(K)**, 4-chamber long-axis **(L)**, increased native T1 **(M)**, decreased post-contrast T1 **(N)** and increased ECV **(O)**. COVID-19, coronavirus disease 2019; LGE, late gadolinium enhancement; ECV, extracellular volume; RV, right ventricular.

As demonstrated in Table 2, compared with healthy controls and risk factor-matched controls, patients with cardiac symptoms after COVID-19 infection had lower left ventricular ejection fractions (LVEFs), peak global circumferential strain (GCS), and peak global longitudinal strain (GLS). Patients with COVID-19 had higher ECV compared with healthy controls. A total of 22 (68.8%) patients were found with non-ischemic pattern of LGE, which were at a mean of 3.6 ± 4.3% of left ventricular myocardium. LGE images in patients after a 1-year recovery from COVID-19 are displayed in Figure 2. Four (19.0%) of risk factor-matched controls also had LGE located in the septum or RV insertion point.

**TABLE 2:** Cardiovascular magnetic resonance (CMR) imaging findings of all participants.

	Healthy controls (*n* = 13)	Risk factor-matched controls (*n* = 21)	COVID-19 patients (*n* = 32)
**Function**			
LVEF (%)	60.1 ± 7.1	59.5 ± 6.8	54.0 ± 5.1^#^^$^
LVEDV (ml)	79.6 ± 12.0	74.8 ± 12.1	76.7 ± 11.6
LVESV (ml)	31.7 ± 6.7	30.4 ± 7.7	35.5 ± 7.7^$^
LVEDV indexed (ml/m^2^)	44.9 ± 10.2	43.3 ± 8.6	45.3 ± 8.4
LVESV indexed (ml/m^2^)	19.1 ± 5.1	18.2 ± 4.7	21.0 ± 5.3
LV mass indexed (g/m^2^)	31.9 ± 7.6	30.7 ± 8.5	26.8 ± 5.5^#^^$^
RVEF (%)	38.8 ± 11.7	39.6 ± 12.6	34.8 ± 11.0
RVEDV (ml)	75.1 ± 17.6	68.8 ± 19.6	75.2 ± 15.0
RVESV (ml)	46.2 ± 15.0	42.6 ± 16.9	48.7 ± 11.4
RVEDV indexed (ml/m^2^)	44.0 ± 10.1	39.3 ± 9.2	44.0 ± 7.8
RVESV indexed (ml/m^2^)	27.1 ± 8.9	23.7 ± 8.5	28.5 ± 6.1^$^
**Strain**			
Radial (%)	39.5 ± 13.0	36.8 ± 9.9	31.7 ± 4.9
Circumferential (%)	–21.1 ± 3.5	–20.4 ± 3.0	–18.8 ± 1.8^#^^$^
Longitudinal (%)	–19.4 ± 2.1	–19.6 ± 2.3	–17.4 ± 2.2^#^^$^
**Mapping**			
T1 (ms)	1206.3 ± 36.0	1236.7 ± 49.1	1219.4 ± 41.2
T2 (ms)	39.7 ± 2.0	40.0 ± 2.6	39.8 ± 3.0
ECV (%)	24.5 ± 1.6	25.1 ± 2.7	26.4 ± 2.0^#^
**LGE**			
LGE, *n* (*%*)	0 (0)	4 (19.0)	22 (68.8)^#^^$^
LGE (%)	0.0 ± 0.0	0.8 ± 1.8	3.6 ± 4.3^#^^$^
LGE segments (*n*)	0.0 ± 0.0	0.7 ± 1.5	5.1 ± 4.1^#^^$^
**LGE localization**			
RV insertion point, *n* (*%*)	–	2 (9.5)	15 (46.9)^#^^$^
Septal, *n* (*%*)	–	2 (9.5)	21 (65.6)^#^^$^
Lateral wall, *n* (*%*)	–	0 (0)	8 (25.0)^#^^$^
**LGE distribution**			
Linear, *n* (*%*)	–	2 (9.5)	20 (62.5)^#^^$^
Patchy, *n* (*%*)	–	2 (9.5)	15 (46.9) ^#^^$^
Diffuse, *n* (*%*)	–	–	–
**LGE pattern**			
Sub-epicardial, *n* (*%*)	–	2 (9.5)	20 (62.5)^#^^$^
Mid-wall, *n* (*%*)	–	2 (9.5)	15 (46.9)^#^^$^
Sub-endocardial, *n* (*%*)	–	–	–

Continuous data are presented as mean ± SD and categorical variables are summarized as percentage in parentheses.

LVEF, left ventricular ejection fraction; LVEDV, left ventricular end-diastolic volume; LVESV, left ventricular end-systolic volume; LV, left ventricular; RVEF, right ventricular ejection fraction; RVEDV, right ventricular end-diastolic volume; RVESV, right ventricular end-systolic volume; ECV, extracellular volume; LGE, late gadolinium enhancement; RV, right ventricular.

*p < 0.05, healthy controls vs. risk factor-matched controls.

^#^*p* < 0.05, healthy controls vs. COVID-19 patients.

^$^*p* < 0.05, risk factor-matched controls vs. COVID-19 patients.

### Subgroup cardiovascular magnetic resonance analysis

**FIGURE 3:**
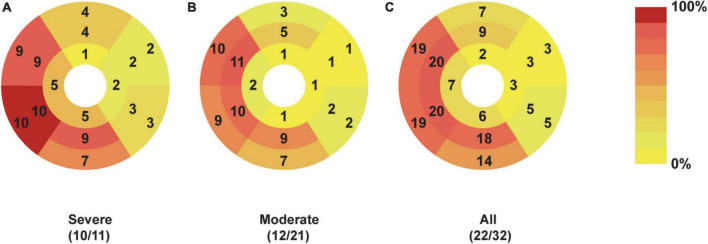
Dominant location and distribution of myocardial LGE segments in patients recovered from COVID-19. The number and frequency of myocardial LGEs based on the AHA 16 segments’ model in patients with severe **(A)**, moderate **(B)**, and all **(C)** COVID-19 were displayed. There were 32 patients enrolled, including 11 patients with severe and 21 patients with moderate COVID-19. Moreover, positive LGEs were found in 22 patients, including 10 patients with severe COVID-19 and 12 patients with moderate COVID-19. AHA, American Heart Association; COVID-19, Coronavirus Disease-2019; LGE, late gadolinium enhancement.

Table 3 shows the comparison of patients with severe and moderate COVID-19. The prevalence of LGE was higher in patients with severe COVID-19 (90.9%) compared with patients with moderate COVID-19 (57.1%), though the difference was not statistically significant (*p* = 0.106). Larger scar volume was found in patients with severe COVID-19, compared with patients with moderate COVID-19 (6.5 ± 5.6% vs. 2.1 ± 2.4%, *p* = 0.009). Patients with severe COVID-19 had more segments involved than patients with moderate COVID-19 (7.7 ± 3.5 vs. 3.9 ± 3.8, *p* = 0.005). LGE showed preponderance for: (1) septal location (52.4 and 90.9% for moderate and severe COVID-19, respectively), (2) linear distribution (52.4 and 81.8% for moderate and severe COVID-19, respectively), and (3) mid-wall pattern (52.4 and 81.8% for moderate and severe COVID-19, respectively). Distribution of myocardial LGE in patients with COVID-19 is shown in Figure 3. Demographic characteristics of patients with moderate and severe COVID-19 symptoms are shown in Supplementary material.

**TABLE 3:** Cardiovascular magnetic resonance (CMR) imaging findings of moderate and severe subgroups.

	Moderate (*n* = 21)	Severe (*n* = 11)	*P*-value
**Function**			
LVEF (%)	53.5 ± 5.0	55.1 ± 5.4	0.413
LVEDV (ml)	77.5 ± 13.6	75.3 ± 6.5	0.545
LVESV (ml)	36.3 ± 8.5	33.9 ± 5.8	0.254
LVEDV indexed (ml/m^2^)	46.2 ± 9.7	43.6 ± 5.3	0.481
LVESV indexed (ml/m^2^)	21.6 ± 5.9	19.6 ± 3.9	0.389
LV mass indexed (g/m^2^)	25.9 ± 5.6	28.4 ± 5.1	0.113
RVEF (%)	32.9 ± 10.7	38.3 ± 11.0	0.389
RVEDV (ml)	72.7 ± 12.7	80.0 ± 18.4	0.257
RVESV (ml)	48.5 ± 10.5	49.1 ± 13.5	0.886
RVEDV indexed (ml/m^2^)	43.2 ± 8.1	45.4 ± 7.3	0.444
RVESV indexed (ml/m^2^)	28.8 ± 6.2	27.9 ± 6.3	0.721
**Strain**			
Radial (%)	32.0 ± 5.7	31.2 ± 3.1	0.617
Circumferential (%)	–18.8 ± 2.1	–18.8 ± 1.1	0.896
Longitudinal (%)	–18.0 ± 2.1	–16.3 ± 2.1	0.029
**Mapping**			
T1 (ms)	1218.2 ± 36.6	1221.7 ± 50.7	0.821
T2 (ms)	39.8 ± 3.0	39.9 ± 3.1	0.949
ECV (%)	26.4 ± 1.5	26.4 ± 2.7	0.95
**LGE**			
LGE, *n* (*%*)	12 (57.1)	10 (90.9)	0.106
LGE (%)	2.1 ± 2.4	6.5 ± 5.6	**0.009**
LGE segments (*n*)	3.9 ± 3.8	7.7 ± 3.5	**0.005**
**LGE localization**			
RV insertion point, *n* (*%*)	6 (28.6)	9 (81.8)	**0.004**
Septal, *n* (*%*)	11 (52.4)	10 (90.9)	**0.048**
Lateral wall, *n* (*%*)	4 (19.0)	4 (36.4)	0.397
**LGE distribution**			
Linear, *n* (*%*)	11 (52.4)	9 (81.8)	0.139
Patchy, *n* (*%*)	6 (28.6)	9 (81.8)	**0.004**
Diffuse, *n* (*%*)	–	–	–
**LGE pattern**			
Sub-epicardial, *n* (*%*)	7 (33.3)	8 (72.7)	**0.034**
Mid-wall, *n* (*%*)	11 (52.4)	9 (81.8)	0.139
Sub-endocardial, *n* (*%*)	–	–	–

Continuous data are presented as mean ± SD and categorical variables are summarized as percentage in parentheses. Bold values indicate *p* < 0.05. LVEF, left ventricular ejection fraction; LVEDV, left ventricular end-diastolic volume; LVESV, left ventricular end-systolic volume; LV, left ventricular; RVEF, right ventricular ejection fraction; RVEDV, right ventricular end-diastolic volume; RVESV, right ventricular end-systolic volume; ECV, extracellular volume; LGE, late gadolinium enhancement; RV, right ventricular.

### Inter-observer agreements

Table 4 shows inter-observer agreements were all excellent concerning LGE location (all kappa value ≥0.839, all *p* < 0.001), distribution (all kappa value ≥0.871, all *p* < 0.001), and pattern (all kappa value ≥0.871, all *p* < 0.001).

**TABLE 4:** Interobserver agreement of late gadolinium enhancement (LGE) location, distribution, and pattern.

	COVID-19 patients (*n* = 32)		
	Kappa	95% CI	*P*-value
**LGE localization**			
RV insertion point, *n* (*%*)	0.937	0.815–1.000	**<0.001**
Septal, *n* (*%*)	0.932	0.801–1.000	**<0.001**
Lateral wall, *n* (*%*)	0.839	0.674–1.000	**<0.001**
**LGE distribution**			
Linear, *n* (*%*)	0.871	0.708–1.000	**<0.001**
Patchy, *n* (*%*)	0.875	0.706–1.000	**<0.001**
Diffuse, *n* (*%*)	–	–	–
**LGE pattern**			
Sub-epicardial, *n* (*%*)	0.938	0.818–1.000	**<0.001**
Mid-wall, *n* (*%*)	0.871	0.708–1.000	**<0.001**
Sub-endocardial, *n* (*%*)	–	–	–

Kappa analysis was performed in evaluating the agreement of two readers in LGE location, distribution, and pattern. Kappa > 0.80 was considered as excellent agreement. Bold values indicate *p* < 0.05. LGE, late gadolinium enhancement; RV, right ventricular; CI, confidence interval.

## Discussion

Decreased LVEF and GLS, increased ECV, and small volume of LGE were observed in patients with persistent cardiac symptoms 1 year after recovery from severe or moderate COVID-19, compared with healthy controls. A higher percentage of LGE volume was found in patients with severe COVID-19 compared with patients with moderate COVID-19. Of note, the LGE lesions of patients recovered from COVID-19 were predominately located in the septal mid-wall or RV insertion point.

Pathologic results and CMR imaging confirmed cardiac involvement in patients with COVID-19. Myocardial interstitial infiltrates by mononuclear inflammatory cells were reported in a postmortem examination of a patient who died from COVID-19, while acute myocarditis was observed in the endomyocardial biopsy of a patient who recovered from COVID-19 (4, 23). Huang et al., analyzing CMR images, found 15 patients with edema or LGE 2 months after discharge from the hospital (8). Puntmann et al. demonstrated ongoing myocardial inflammation and LGE in patients with COVID-19 independent of whether they recovered at home or required hospitalization (17).

There was a high prevalence of myocardial fibrosis (68.8%) in our study, especially in patients with severe COVID-19. The prevalence of LGE lesions ranged from 4 to 69% in previous studies (24, 25). The relationship between the severity of pneumonia and cardiac involvement is controversial. Two months after infection, patients with severe COVID-19 had higher myocardial T2 values than patients with moderate COVID-19 (26). Additionally, 3 months after infection, persistent RV dysfunction was observed in patients with severe COVID-19 (27). However, no relationship between pneumonia severity and myocardial involvement was demonstrated by Puntmann et al. (17) and Myhre et al. (28). Possible explanations for these discrepancies may relate to differences in the time interval from acute infection to CMR examination and the criteria for patient inclusion and exclusion. More studies are needed to settle this controversial issue (29).

The mid-wall LGE lesions were predominantly displayed in patients with COVID-19, which were similar to human herpesvirus 6 myocarditis, dilated cardiomyopathy, and alcohol-induced cardiomyopathy (20, 30, 31), but unlike typical myocarditis (subepicardial enhancement in the lateral wall). In previous reports, fibrosis in the mid-wall of the septum and RV insert point was also observed in patients with COVID-19 (9, 10, 13). The mechanisms for mid-wall fibrosis were the result of multiple factors, including exposure to toxins and pathogens, immunomodulatory disorders, abnormal activation of the renin-angiotensin system, and microvascular ischemia (32, 33). For patients with COVID-19, severe systemic hyperinflammation, increased autoantibody reactivity, and over-activation of the renin-angiotensin system were all identified, which may be related to the myocardial mid-wall fibrosis (34–36). The prognosis of these mid-wall LGE lesions in patients with COVID-19 is unclear. As reported in patients with myocarditis, the presence of mid-wall septal LGE was strongly associated with major adverse cardiovascular events at a median follow-up of 4.7 years (19). Greulich et al. demonstrated that the presence of mid-wall LGE in septal segments was associated with a higher rate of sudden cardiac death at a median follow-up of 10.1 years, which may be due to involvement of the conduction system (21). The human herpesvirus 6 infects cells of the nervous and cardiac conduction systems, which may be related to the septal LGE (30). Longer follow-up studies are needed.

In our study, LGE lesions at the RV insertion point were common in patients with COVID-19, especially those with severe COVID-19. This type of LGE pattern is typically observed in athletes and patients with pulmonary hypertension (37, 38). Focal LGE in the insertion point is related to mechanical wall stress and elevated RV afterload (38). The presence of LGE at the RV insertion point was more prevalent in patients with COVID-19 (46.9%) than that in risk factor-matched controls (9.5%). The prevalence of LGE in population-based cohort was 29.0% in the study of Barbier et al. (39) and 7.9% in the study of Turkbey et al. (40). Such a high prevalence of RV insertion point involvement may be related to pulmonary hypertension due to vasoconstriction, vascular thrombosis, vascular remodeling, and hypoxia during the patient’s hospitalization (41). LGE lesions at RV insertion point were also displayed in previous studies in patients with COVID-19 (8, 13). However, this finding needs to be confirmed with CMR before and during the acute phase of COVID-19.

Compared with healthy subjects, there was elevated ECV in patients that recovered from COVID-19, but no significant difference between the severe and moderate pneumonia groups. Increased ECV suggests diffuse myocardial interstitial fibrosis in patients with COVID-19, and LGE reflected focal replaced fibrosis (32). Surprisingly, there was no statistically significant correlation between LGE and ECV, which was consistent with the non-statistical difference in ECV between LGE positive and negative groups (13). Therefore, we inferred that diffuse interstitial and focal fibrosis might be two separate myocardial pathologic changes caused by COVID-19 infection. However, large sample size and baseline CMR are needed to verify this hypothesis. ECV was derived from the ratio of T1 signal values and is sensitive to expanded extracellular space (42). Equivalent native T1 and increased ECV were observed between healthy controls and patients, which was consistent with Li et al., but inconsistent with the findings of increased T1 values from Huang et al. and Puntmann et al. (5, 8, 17). This difference may be due to the varying lengths of time between COVID-19 infection and when CMR exams were performed. After 1 or 2 months of recovery, myocardial edema was observed (8, 17). Native T1, a composite indicator of both intracellular and extracellular components, was reportedly more sensitive to other tissue characteristics (fat content, iron levels, and edema) than the extracellular space (42, 43). The importance of this results needs long-term follow-up (5).

We observed decreased GLS in our participants, as previously reported (5). In the acute phase, decreased GLS was related to increased mortality of hospitalized patients. The impact of decreased GLS on long-term prognosis remains unknown and requires further study. Petersen et al. (44) reported subclinical involvement after COVID-19. The clinical significance of findings in this study needs to be further verified.

Our study had some limitations. Firstly, this study lacked baseline CMR examinations before and during acute infection, which limited the evaluation of cardiac involvement progress. Secondly, the number of patients and controls included was small. More patients are required in future studies to eliminate overfitting and random biases. Finally, the echocardiography was read by different readers from different hospitals. The inter-reader variability could potentially affect the accuracy of echocardiography results. Future study may need to evaluate this variability in the diagnosis of COVID-19 patients.

## Conclusion

This 1-year follow-up study revealed that patients with persistent cardiac symptoms after recovering from COVID-19 infection showed decreased cardiac function and increased ECV compared with healthy controls. Compared with risk factor-matched controls, patients with COVID-19 had a higher prevalence of LGE compared with risk factor-matched controls, which was primarily located in the mid-wall septum or RV insertion point. And patients with severe COVID-19 had greater LGE extent, compared with patients with moderate COVID-19.

## Data availability statement

The original contributions presented in this study are included in the article/Supplementary material, further inquiries can be directed to the corresponding author.

## Ethics statement

The studies involving human participants were reviewed and approved by the Ethical Committee of the Tongji Medical College, Huazhong University of Science and Technology (2021-S114). The patients/participants provided their written informed consent to participate in this study.

## Author contributions

JQ, PZ, and LH conceived and designed the study. JQ, PZ, and JL collected clinical and CMR data. JQ, PZ, and LX analyzed the data. XZ provided technical support. JQ performed the statistical analysis and drafted the manuscript. PZ, LH, XM, and XZ provided critical revision of the manuscript. All authors contributed to the article and approved the submitted version.
