# Supplementary material for: Cardiac involvement in patients 1 year after recovery from moderate and severe COVID-19 infections
Source: Front Cardiovasc Med. 2022 Oct 26;9:1009637. doi: 10.3389/fcvm.2022.1009637 (PMC9646443; doi:10.3389/fcvm.2022.1009637)
Supplement: Supplementary file 1 [file Table_1.docx]

**Supplement Table 1. Demographic and clinical information of moderate and severe subgroups.**

|  | Moderate  (*n* = 21) | Severe  (*n* = 11) | *p* value |
| --- | --- | --- | --- |
| Age (years) | 47.6 ± 10.0 | 50.6 ± 11.7 | 0.449 |
| Male, *n* (*%*) | 3 (14.3) | 4 (36.4) | 0.197 |
| Time between acute COVID-19 infection and CMR (days) | 460.8 ± 15.7 | 463.9 ± 22.0 | 0.649 |
| BMI (kg/m^2^) | 24.1 ± 2.5 | 23.9 ± 3.4 | 0.828 |
| Systolic BP (mmHg) | 118.1 ± 12.5 | 119.8 ± 14.6 | 0.721 |
| Diastolic BP (mmHg) | 77.1 ± 10.2 | 78.1 ± 14.6 | 0.823 |
| Smoke, *n* (*%*) | 3 (14.3) | 2 (18.2) | 0.999 |
| Drink, *n* (*%*) | 1 (4.8) | 2 (18.2) | 0.266 |
| Hypertension, *n* (*%*) | 1 (4.8) | 6 (54.5) | **0.003** |
| Diabetes, *n* (*%*) | 2 (9.5) | 2 (18.2) | 0.593 |
| Hypercholesterolemia, *n* (*%*) | 4 (19.0) | 6 (54.5) | 0.056 |
| Hct (%) | 40.7 ± 4.0 | 41.4 ± 2.5 | 0.223 |
| Elevated troponin, n (%) ^a^ | 7 (36.8) | 4 (36.4) | 0.999 |
| Abnormal echocardiography, n (%) ^b^ | 6 (33.3) | 5 (45.5) | 0.696 |
| Abnormal ECG, n (%) ^c^ | 9 (47.4) | 5 (55.6) | 0.999 |

Continuous data are presented as mean ± SD and categorical variables are summarized as percentage in parentheses. Bold values indicate *p* < 0.05.

BMI, body mass index; BP, blood pressure; CMR, cardiovascular magnetic resonance; COVID-19, coronavirus disease 2019; Hct, hematocrit.

^a^ Troponin tests were performed in 19 patients with moderate COVID-19 and 11 patients with severe COVID-19.

^b^ Echocardiography was performed in 18 patients with moderate COVID-19 and 11 patients with severe COVID-19.

^c^ ECG was performed in 19 patients with moderate COVID-19 and 9 patients with severe COVID-19.
